# Associations of Racial and Ethnic Category, Age, Comorbidities, and Socioeconomic Factors on Concordance to NCCN Guidelines for Patients With High-Risk Biliary Tract Cancers After Surgery

**DOI:** 10.3389/fonc.2022.771688

**Published:** 2022-02-22

**Authors:** Lauren F. Huang, Augustine Hong, Gino Cioffi, Asrar Alahmadi, Tin-Yun Tang, Lee M. Ocuin, Nirav Patil, David L. Bajor, Joel N. Saltzman, Amr Mohamed, Eva Selfridge, Monica Webb Hooper, Jill Barnholtz-Sloan, Richard T. Lee

**Affiliations:** ^1^ Department of Medicine, Case Western Reserve University School of Medicine, Cleveland, OH, United States; ^2^ Department of Medicine, University Hospitals Cleveland Medical Center, Cleveland, OH, United States; ^3^ Department of Population and Quantitative Health Sciences, Case Western Reserve University School of Medicine, Cleveland, OH, United States; ^4^ Department of Internal Medicine, Ohio State University James Thoracic Oncology Center, Comprehensive Cancer Center, Columbus, OH, United States; ^5^ Division of Cancer Medicine, University of Texas MD Anderson Cancer Center, Houston, TX, United States; ^6^ Department of Surgery, University Hospitals Cleveland Medical Center, Cleveland, OH, United States; ^7^ Department of Psychology, Case Comprehensive Cancer Center, Cleveland, OH, United States; ^8^ Department of Medicine, Case Western Reserve University Comprehensive Cancer Center, Cleveland, OH, United States

**Keywords:** bile tract cancer, cholangiocarcinoma, health disparities, concordance, race

## Abstract

**Background:**

Biliary tract cancers (BTC) have a limited prognosis even for localized cancers, emphasizing the importance of multidisciplinary management. NCCN guidelines recommend adjuvant chemotherapy (CT) +/- radiotherapy (RT) for high-risk disease. We analyzed the association between racial and ethnic category along with other demographic factors and concordance to NCCN guidelines among patients following surgery for high-risk BTC.

**Methods:**

Subjects were identified from the National Cancer Database (NCDB) for BTC patients who underwent surgery and found to have metastatic lymph nodes (LN+) or positive surgical margins (M+) from 2004 to 2015. We defined concordance to NCCN guidelines as receiving surgery + CT +/- RT and non-concordance to the guidelines as surgery +/- RT. Descriptive studies and multivariate logistic regression analysis was performed.

**Results:**

A total of 3,792 patients were identified with approximately half being female (55.4%) and between the ages of 50-69 (52.8%). Most were White (76.3%) followed by Black (10.6%), Hispanic (8.5%), and Asian (5.3%). The BTC included extrahepatic cholangiocarcinoma (CCA) (48.6%), gallbladder cancer (43.5%), and intrahepatic CCA (7.9%). Most patients had an M- resection (71.9%) but also had LN+ disease (88.0%). There were no significant differences between racial groups in disease presentation (histological grade, tumor stage) and surgical outcomes (LN+, M+, hospital readmission, and 90 day post-surgery mortality). Hispanic patients as compared to White patients were less likely to be insured (85.7% vs 96.3%, p<0.001) and less likely to be treated at an academic facility (42.1% vs 52.1%, p=0.008). Overall, almost one-third (29.7%) of patients received non-concordant NCCN guideline care with Hispanic patients having the highest proportion of non-concordance as compared to Whites patients (36.1% vs 28.7%, p=0.029). On multivariate analysis, Hispanic ethnicity (HR=1.51, 95% CI: 1.15-1.99) remained significantly associated with non-concordance to NCCN guidelines.

**Conclusion:**

This study indicates that Hispanic patients with high-risk BTC are significantly less likely to receive NCCN-concordant treatment in comparison to White patients. More research is needed to confirm and understand the observed disparities and guide targeted interventions at the system-level.

## Introduction

Biliary tract cancers (BTCs) are a rare cancer that make up 3% of all adult cancers, with 11,980 new patient cases and 4,310 deaths in 2021 in the United States, although this number does not include intrahepatic cholangiocarcinomas (1, 2). BTCs most commonly present as gallbladder cancer (GBC), but also include extrahepatic cholangiocarcinoma (ECC), intrahepatic cholangiocarcinoma (ICC). The incidence of GBC is higher among women, who have increased susceptibility to the primary GBC risk factors (i.e., gallstone disease and chronic cholecystitis), as well as among individuals residing in Chile, Japan, and northern India (3). A recent 2019 analysis of worldwide trends in mortality from ICC and ECC showed that ICC incidence and mortality have increased in recent years (4). GBC makes up approximately two-thirds of BTCs and remains the most aggressive of the BTCs with the shortest median survival duration (5).

Patients affected by BTCs are usually diagnosed at late stages of disease due to the rapid onset and asymptomatic nature of early disease, or potential misdiagnosis as biliary colic; late diagnosis often results in poor prognosis (6). Surgery remains essential for a curative approach, although data from the SEER registry indicates that the overall 5-year-survival is estimated to be 10% across all patients (7). The postoperative median overall survival estimated to be 18-30 months is based on the BILCAP clinical trial with adjuvant capecitabine now being the standard of care, and patients with positive lymph node status and resection margins were reported to have a worse prognosis (8, 9). National Comprehensive Cancer Network (NCCN) guidelines recommend multimodality therapy for all patients diagnosed with node-positive resected cholangiocarcinoma, specifically systemic chemotherapy with or without targeted radiation (10, 11). Both chemotherapy and chemoradiation have been associated with improved survival outcomes in BTC patients with lymph node-positive disease (12). Furthermore, methods to improve BTC treatment such as tumor genetic profiling have increased over the last decade and led to the approval of IDH1 and FGFR2 inhibitors (13–15). On the other hand, however, addition of certain emerging therapies such as anti-EGFR monoclonal antibodies to existing therapeutic regimens and immunotherapy have not significantly improved overall and progression-free survival (16).

Since cholangiocarcinoma remains such a challenge to the field of oncology due to its poor prognosis and difficult course of treatment, equal access to and receipt of proper treatment is of paramount importance. Although racial disparities in the diagnosis, incidence, and time to treatment of hepatobiliary cancers have been investigated in recent years, there is still limited data on the association of racial and ethnic category in the receipt of NCCN-concordant treatment for resected high-risk BTCs (17). Based on the recently reported disparities in receipt of surgery, quality of lymph node clearance post-resection, and unequal improvements in overall survival and incidence rates of BTCs, our aim was to investigate the associations between race, ethnicity, and socioeconomic factors on the receipt of treatment aligned to NCCN guidelines for high-risk BTC using the National Cancer Database (NCDB). For the purposes of this study, the term concordance indicates the extent to which patients received a treatment that was recommended by NCCN guidelines.

## Methods

### National Cancer Database

The NCDB is a clinical oncology database sponsored by the American College of Surgeons and the American Cancer Society that captures approximately 70% of all cancer diagnoses in the United States annually. The NCDB database collects prospective data from over 1,500 Commission on Cancer accredited facilities, offering information regarding malignant neoplastic diseases, their treatments, and outcomes. After receiving approval from the NCDB, patients diagnosed with BTC were identified (intrahepatic cholangiocarcinoma, extrahepatic cholangiocarcinoma, gallbladder, and primary malignant neoplasm of overlapping sites of biliary tract) using the NCDB Participant User File, and only post-operative patients ≥ 18 years with diagnosis of high-risk BTC were identified by histology code and included in this analysis. The research protocol was submitted to the IRB for review and it was determined that approval was not required to conduct this analysis.

Patients were considered high-risk if they had positive margin (M+) or lymph node metastases (LN+) without distant metastases. Additional data variables were used to further categorize patients based on age, race and ethnicity (non-Hispanic White, non-Hispanic Black, Hispanic, Asian), sex, Charlson-Deyo Comorbidity Index (CCI), treatment type (surgery, chemotherapy, radiation), time of treatment, facility type (academic, integrated network cancer centers, comprehensive community centers, and community centers), insurance type (Medicare, Medicaid, private, none) and estimated household income (<$38,000, $38,000-$47,999, $48,000-$62,999, >=$68,000). Regarding facility types, the academic and integrative network categories were combined, as many academic centers are categorized to be integrative networks if they include multiple sites.

### Statistical Analysis

Univariate analysis of demographic and clinical characteristics between proper and improper treatment was performed using Chi-square test for categorical variables and independent sample t-test or Wilcoxon test for continuous variables. Multivariable analysis for survival was conducted using Cox proportional hazards models. The Kaplan-Meier method was used for univariate survival analysis. Univariate and multivariable odds ratio for Race to receive improper treatment was assessed using logistic regression analysis. Multivariable analysis included Age, Sex, Insurance, Income, Stage, Surgical Margin and Charlson-Deyo score. Patients diagnosed in 2015 were excluded from the database due to insufficient data. SAS version 9.4 was used to perform all analyses. A *P*-value ≤ 0.05 was considered statistically significant.

## Results

### Cohort Demographics

The NCDB included 8,791 cases of BTC from 2004-2014. Based on inclusion criteria for the study, our study identified a total of 3,792 post-operative, high-risk patients diagnosed with BTC. The majority of patients identified were women (55.4%), White non-Hispanic (76.3%), and between the ages of 50-69 years (52.8%). The majority of patients were also insured – Medicare (49.0%), private insurance (38.8%), Medicaid (6.1%), and uninsured (3.5%). One-third of patients (33.6%) were estimated to have a median household income of ≥ $68,000, while 15.4% had an income of <$38,000. Patients were treated at one of four types of facilities: academic/research cancer center (51.3%), comprehensive community cancer program (29.4%), community cancer program (5.4%), and integrated network cancer program (11.8%). See **Table 1**.

**Table 1:** Demographics.

	Total	Concordant	Non-Concordant	P
Overall, n	3792	2667 (70.3%)	1125 (29.7%)	
Age group, n (%)				
<50 years	331 (8.7%)	271 (10.2%)	60 (5.3%)	**<0.0001**
50–69 years	2004 (52.8%)	1548 (58%)	456 (40.5%)	
>70 years	1457 (38.4%)	848 (31.8%)	609 (54.1%)	
Sex, n (%)				
Male	1693 (44.6%)	1220 (45.7%)	473 (42%)	**0.036**
Female	2099 (55.4%)	1447 (54.3%)	652 (58%)	
Race and ethnicity, n (%)				
White	2893 (76.3%)	2062 (77.3%)	831 (73.9%)	**0.029**
Black	376 (9.9%)	255 (9.6%)	121 (10.8%)	
Hispanic	321 (8.5%)	205 (7.7%)	116 (10.3%)	
Asian and Pacific Islander	202 (5.3%)	145 (5.4%)	57 (5.1%)	
Charlson/Deyo Score, n (%)				
0	2778 (73.3%)	1992 (74.7%)	786 (69.9%)	**0.0003**
1	805 (21.2%)	547 (20.5%)	258 (22.9%)	
2	143 (3.8%)	95 (3.6%)	48 (4.3%)	
≥3	66 (1.7%)	33 (1.2%)	33 (2.9%)	
Insurance status, n (%)				
Not insured	133 (3.5%)	92 (3.5%)	41 (3.7%)	0.735
Insured	3608 (95.1%)	2545 (96.5%)	1063 (96.3%)	
Median income—regional, n (%)				
<$38,000	583 (15.4%)	390 (14.8%)	193 (17.5%)	**0.001**
$38,000–$47,999	841 (22.2%)	563 (21.4%)	278 (25.2%)	
$48,000–$62,999	1038 (27.4%)	735 (27.9%)	303 (27.5%)	
≥$68,000	1273 (33.6%)	944 (35.9%)	329 (29.8%)	
Facility type, n (%)				
Community cancer	204 (5.4%)	132 (5.1%)	72 (6.5%)	**0.054**
Comprehensive community cancer	1116 (29.4%)	761 (29.2%)	355 (32%)	
Academic/research	1944 (51.3%)	1397 (53.6%)	547 (49.3%)	
Integrated Network Cancer	449 (11.8%)	314 (12.1%)	135 (12.2%)	
Histology grade, n (%)				
Well differentiated	347 (9.2%)	213 (8.5%)	134 (12.5%)	**0.0004**
Moderately differentiated	1756 (46.3%)	1265 (50.5%)	491 (45.9%)	
Poorly differentiated/undifferentiated	1473 (38.8%)	1029 (41%)	444 (41.5%)	
Location, n (%)				**<0.0001**
Gall bladder	1649 (43.5%)	1042 (39.1%)	607 (54%)	
Extrahepatic	1844 (48.6%)	1423 (53.4%)	421 (37.4%)	
Intrahepatic	299 (7.9%)	202 (7.6%)	97 (8.6%)	
Tumor stage, n (%)				
1	119 (3.1%)	52 (2%)	67 (6.1%)	**<0.0001**
2	1769 (46.7%)	1244 (47.4%)	525 (47.5%)	
3	1229 (32.4%)	879 (33.5%)	350 (31.6%)	
4	614 (16.2%)	450 (17.1%)	164, (14.8%)	
Lymphadenectomy, n (%)				
No	251 (6.6%)	140 (5.3%)	111 (10%)	**<0.0001**
Yes	3502 (92.4%)	2504 (94.7%)	998 (90%)	
Positive lymph nodes, n (%)				
Negative	455 (12%)	260 (9.7%)	195 (17.3%)	**<0.0001**
Positive	3337 (88%)	2407 (90.3%)	930 (82.7%)	
Surgical margins, n (%)				
R0	2726 (71.9%)	1967 (73.8%)	759,(67.5%)	**0.0004**
R1	969 (25.6%)	634 (23.8%)	335 (29.8%)	
R2	97 (2.6%)	66 (2.5%)	31 (2.8%)	
Treatment group, n (%)				
Surgery only	1049 (27.7%)	0	1,049 (93.2%)	**<0.0001**
Surgery and radiation	76 (2.0%)	0	76 (6.8%)	
Surgery and chemotherapy	1247 (32.9%)	1,247 (46.8%)	0	
Surgery, chemotherapy, and radiation	1420 (37.4%)	1,420 (53.2%)	0	

Bold indicates p < 0.05.

Almost half of patients had moderately differentiated histological grade (46.3%) and over one-third (38.8%) of patients had poorly differentiated/undifferentiated histological grade. Forty-three percent of patients were diagnosed with gallbladder cancer, 38.8% of patients were diagnosed with extrahepatic duct cancer, and 7.9% of patients were diagnosed with intrahepatic duct cancer. The majority of BTCs were assigned a tumor stage of two (T2) (46.7%), followed by T3 (32.4%), and T4 (16.2%). The majority of patients were LN+ (88%) and M- (71.9%). After surgery, patients received one of the following approaches: surveillance (27.7%), chemotherapy (32.9%), radiation (2%), and a combination of chemotherapy and radiation (37.4%).

### Survival Analysis

Analysis of the NCDB database revealed several factors that were associated with overall survival. Patients who did not receive adjuvant chemotherapy treatment following surgery were 53% less likely to survive (HR 1.53, 1.39-1.68; p<0.0001), and patients older than 70 years old were 36% less likely to survive (HR 1.36, 1.11-1.66; p = 0.002). A higher CCI was also correlated with a worse prognosis, specifically CCI=1 (HR 1.20, 1.08-1.32; p<0.0001), CCI=2 (HR 1.25, 1.02-1.54; p = 0.033), and a CCI ≥ 3 (HR 1.33, 0.99-1.8; p = 0.058). Furthermore, patients treated at community cancer programs (CCP) or comprehensive community cancer programs (CCCP) had worse outcomes (CCP: HR 1.3, 1.09-1.56; p = 0.004 and CCCP: HR 1.19, 1.08-1.31; p<.0001). See **Table 2**.

**Table 2:** Survival Analysis, Adjusted Cox Proportional Hazards Model.

	Adjusted Hazard Ratio (95% CI)	P
Age group		
<50 years	Ref	
50–69 years	1.17 (0.96–1.42)	0.116
>70 years	**1.36** (1.11–1.66)	**0.002**
Sex		
Female	Ref	
Male	1.04 (0.95–1.14)	0.362
Race and ethnicity, n (%)		
White	Ref	
Asian and Pacific Islander	0.91 (0.75–1.1)	0.332
Black	0.94 (0.82–1.09)	0.430
Hispanic	0.9 (0.76–1.06)	0.191
Charlson/Deyo Score		
0	Ref	
1	**1.2** (1.08–1.32)	**<0.0001**
2	**1.25** (1.02–1.54)	**0.033**
>=3	1.33 (0.99–1.8)	0.058
Insurance status		
Insured	Ref	
Not insured	1.09 (0.86–1.39)	0.478
Median income—regional		
<$38,000	0.96 (0.84–1.09)	0.494
$38,000–$47,999	1.07 (0.96–1.2)	0.232
$48,000–$62,999	1.02 (0.91–1.13)	0.769
>=$68,000	Ref	
Facility type		
Academic/research	Ref	
Community cancer	**1.3** (1.09–1.56)	**0.004**
Comprehensive Community Cancer	**1.19** (1.08–1.31)	**<0.0001**
Integrated Network Cancer	1.04 (0.91–1.19)	0.562
Histology grade		
Well differentiated	Ref	
Moderately differentiated	**1.31** (1.12–1.52)	**0.001**
Poorly differentiated/undifferentiated	**1.57** (1.35–1.84)	**<0.0001**
Location		
Gall bladder	Ref	
Extrahepatic	**0.75** (0.67–0.83)	**<0.0001**
Intrahepatic	**0.83** (0.7–0.99)	**0.037**
Tumor stage		
1	Ref	
2	1.13 (0.88–1.46)	0.346
3	1.18 (0.91–1.52)	0.224
4	**1.84** (1.4–2.41)	**<0.0001**
Positive lymph nodes		
Negative	Ref	
Positive	**1.51** (1.28–1.77)	**<0.0001**
Surgical margins		
R0	Ref	
R1	**1.79** (1.6–2)	**<0.0001**
R2	**2.39** (1.82–3.13)	**<0.0001**
Treatment		
Proper	Ref	
Improper	**1.53** (1.39–1.68)	**<0.0001**

Bold indicates p < 0.05.

ICC (HR 0.83, 0.7-0.9; p=0.037) and ECC (HR 0.75, 0.67-0.83; p<0.0001) were associated with better overall survival than those patients with GBC. T4 and positive lymph node status were associated with decreased survival (HR 1.84, 1.4-2.41; p<0.0001 and HR 1.51, 1.28-1.77; p<0.0001, respectively). Patients with poorly differentiated/undifferentiated disease were 57% less likely to survive (HR 1.57, 1.35-1.84, p<.0001), while patients with moderately differentiated disease were 31% less likely to survive (HR 1.31, 1.12-1.52, p=0.001). Having a positive margin was significantly associated with decreased survival (R1: 1.79 (1.6-2), p<0.0001; R2: 2.39 (1.82-3.13), p<0.0001). Lastly, patients who received improper treatment, defined as non-concordance to NCCN guidelines (i.e., did not receive adjuvant chemotherapy) were also associated with worse survival (HR 1.53 (1.39-1.68), p<0.0001).

### NCCN Concordance for Adjuvant Chemotherapy and Health Disparities

The majority of patients with high-risk BTCs received NCCN-concordant treatment (70.3%). Women and patients >70 years-old were less likely to receive concordant NCCN guideline treatment as compared to men and patients <50 years-old, (31.1% vs 27.9%, p=0.036) and (41.8% vs. 18.1%, p<0.0001) respectively. Patients with a CCI≥3 were less likely to receive adjuvant chemotherapy in comparison to those with no comorbidities, (50.0% vs 28.3%, p=0.0003). Patients from lower median income areas (<$48K) were also less likely to receive proper adjuvant treatment as compared to those from high income regions (≥68K) (33.1% vs 25.8%, p=0.001). Notably, racial and ethnic minority patients were more likely to receive non-NCCN guideline concordant care, specifically Hispanic (36.1%) and Black patients (32.2%) as compared to White patients (28.7%, p=0.029) (**Table 3**). Multivariate analysis of patients showed that Hispanic patients were 51% more likely to receive non-concordant NCCN treatment when compared to White patients (p=0.003) (**Table 4**).

**Table 3:** Receiving NCCN-Concordant Adjuvant Treatment by Race.

	Concordant	Non-Concordant	P
Overall, n	2667 (70.3%)	1125 (29.7%)	
Race and ethnicity			**0.029**
White	2062 (71.3%)	831 (28.7%)	
Black	255 (67.8%)	121 (32.1%)	
Hispanic	205 (63.9%)	116 (36.1%)	
Asian & Pacific Islander	145 (71.8%)	57 (28.2%)	

Bold indicates p < 0.05.

**Table 4:** Multivariate Logistic Regression for Receiving NCCN-Concordant Adjuvant Treatment.

	Univariate, OR (95% CI)	P	Multivariable,* HR (95% CI)	P
Race and ethnicity				
White	Ref		Ref	
Asian and Pacific Islander	0.98 (0.711–1.339)	0.878	1.06 (0.752–1.49)	0.746
Black non-Hispanic	1.18 (0.935–1.483)	0.166	1.24 (0.957–1.601)	0.105
Hispanic	**1.40** (1.103–1.788)	**0.006**	**1.51** (1.154–1.977)	**0.003**

*Adjusted for Age, Sex, Insurance, Income, Stage, Surgical Margins, and Charlson/Deyo Score. Bold indicates p < 0.05.

## Discussion

Our analysis of the NCDB database is the first to reveal that Hispanics are less likely to receive NCCN guideline-concordant treatment for high-risk BTC. Consistent with more recent literature, not receiving adjuvant therapy for high-risk BTC correlates with worse outcomes (8, 18). Overall, 29.7% of patients did not receive treatment in accordance to NCCN guidelines, with Hispanic patients (36.1%) having the highest proportion of non-concordant care — approximately a 20% increase above White patients. On multivariate analysis, Hispanic ethnicity remained significantly related to receiving treatment that was non-concordant with NCCN guidelines.

It should be noted that the term “adherence” indicates the extent to which patients voluntarily followed through with NCCN guideline-concordant treatment prescribed by their health care providers. This is in contrast to the term “compliance,” which suggests that the patient is passively following the practitioner’s orders and implies lack of partnership between provider and patient in determining the patient’s treatment plan (19). We are currently unable to determine if concordance to NCCN guidelines or lack thereof is due to factors associated with clinician, the patient, or both, as adherence is a very complex and multifaceted issue that is beyond the scope of this study. Potential factors that may influence NCCN-concordant treatment for BTCs may involve clinician bias, healthcare provider opinion on eligibility of patients to receive NCCN recommended treatment, hesitancy toward chemotherapy treatment, and logistical abilities of the healthcare system as well as the patient and their loved ones to maintain a prescribed treatment regimen.

There is existing evidence of racial and ethnic and socioeconomic disparities in the treatment of other hepatobiliary cancers. In a recent evaluation of GBC patients from the 2000–2013 SEER registry, Black patients were less likely to receive surgery when compared to White patients. Furthermore, Hispanic patients were less likely to have optimal lymph node clearance post-resection when compared to White patients (20). In another evaluation of the 2001–2012 SEER database, overall survival in GBC has improved in all racial and ethnic groups, except for Hispanic and Black patients. Hispanic individuals also exhibit the highest incidence rates of gallbladder cancer, and incidence rates in Black individuals are on the rise (21). Additionally, a recent study analyzing 13,965 patients from the NCDB concluded that the odds of CCA in admitted patients were higher for the Hispanic population (22). The study also noted that Hispanic patients did not display different propensity-matched odds of inpatient mortality, morbidity, hospital length of stay, or resource utilization compared to non-Hispanic patients. In a study analyzing the Nationwide Inpatient Sample database between 2005-2014, patients that received surgery for intrahepatic cholangiocarcinoma were typically White, in a younger age cohort, privately insured, less burdened by comorbidities, and more likely to be treated in hospitals located in urban rather than rural geographical areas (23). Furthermore, a recent study identified 12,095 patients with cholangiocarcinoma through the NCDB, concluding that Black patients had lower odds of receiving surgery when compared to White patients (odds ratio [OR] 0.66l; P <.001). The same study also concluded that there were no racial and ethnic or socioeconomic differences in receipt of multimodality therapy once patients accessed surgical care, however (17). A study using 1995-2014 SEER registry showed that although Black patients had a lower incidence of intrahepatic CCA (iCCA), these patients had higher mortality than their White counterparts. This study also highlighted a higher incidence of iCCA amongst Hispanic patients as well as worse all-cause mortality and iCCA specific mortality in Hispanic patients compared to non-Hispanic patients (24). However, our study provides additional findings regarding ethnic disparities in the Hispanic population that have not yet been mentioned in the literature. These differences are likely due to our focused inclusion criteria for analysis in the NCDB, which specifically included BTC patients who underwent surgery and were found to have metastatic lymph nodes or positive surgical margins from 2004 to 2014.

Hispanic persons may receive less appropriate care for several reasons. Notably, our data revealed that Hispanic patients were less likely to be insured (with approximately 85% coverage rather than 95% in other races/ethnicities), less likely to be treated at an academic facility, and had additional comorbidities when compared to White patients. These findings are significant in the context of available disparities research in other gastrointestinal cancers. The significance of insurance coverage was also shown in a previous study for HCC which noted that insurance status had the most profound effect on the likelihood of surgical treatment in patients with HCC (25). In a study investigating pancreatic adenocarcinoma, uninsured patients and patients with Medicaid insurance were less likely to receive adequate treatment and surgery than patients with private insurance, although in our study we did not find any significant association with insurance status (26). Another study investigating colorectal cancer revealed that underinsured individuals also have increased comorbidities and are less likely to have access to quality care as a result (27).

Although the current study lacks the ability to provide concrete solutions to the issues presented, we introduce the discovered disparities with optimism, as identification of an issue is the first step to improvement. The findings of this study could provide a basis for future disparities research to be conducted, with the end goal being equity in clinical evaluation and treatment of patients with BTC regardless of race, ethnicity, Charlson-Deyo Comorbidity Index, or socioeconomic factors. Potential first steps to alleviating this problem in healthcare would be a study evaluating the deductive reasoning process oncologists utilize when prescribing treatments for patients diagnosed with BTCs. Another potential approach to addressing the presented disparities would be a study evaluating the potential implicit biases and attitudes toward race, ethnicity, socioeconomic factors, and comorbidities in oncologists involved in the diagnosis and treatment of BTCs. This study does not capture the complexity of decision-making and the role of patient versus provider in lack of concordance to NCCN guidelines. Future studies are needed to explore these issues by evaluating factors influencing both providers and patients.

The limitations of our study mainly involve pre-existing limitations in the NCDB database. While the NCDB database represents more than 70% of newly diagnosed cancer cases nationwide (including over 34 million historical records), 30% of patient data is still missing. Second, the NCDB database lacked information regarding the functional status of patients and severity of co-morbidities, which are important factors in deciding whether to recommend adjuvant chemotherapy. Furthermore, utilizing a retrospective database automatically integrated selection bias into our study. The patients included in the NCDB have already overcome several barriers to healthcare access; thus, the dataset provided by NCDB inherently excluded patients who did not have the resources or opportunity to be treated at a participating cancer center. Additionally, distance to cancer centers and transportation are not captured in the NCDB but may play an important role in treatment location. It is also of paramount importance to acknowledge that this study was unable to capture the multitude of additional factors that contribute to disparities. Finally, the current study is not able to understand the absolute root cause of these racial disparities, and as a result we are unable to offer tangible solutions that will benefit Hispanic and Black patients.

In summary, this study revealed that Hispanic patients with high-risk BTC were significantly more likely to receive non-concordant care when compared to White patients in regards to NCCN guidelines. These findings are consistent with recent studies revealing additional disparities in Hispanic and Black persons related to the diagnosis, incidence, and time to treatment of hepatobiliary cancers. The findings of this study could help identify high-risk BTC patients who are most likely to not receive adjuvant treatment as recommended by NCCN guidelines, and more importantly, patients who are at an increased risk of suffering from higher rates of morbidity and mortality due to unequal access to and receipt of cancer treatment. Additional research is needed to confirm and understand the observed disparities and guide targeted interventions, with the ultimate goal of eliminating disparities in healthcare.

## Data Availability Statement

The datasets presented in this article are not readily available because the data that support the findings of this study are available from the American College of Surgeons—Commission on Cancer. Restrictions apply to the availability of these data, which were used under license for this study. Data are available from the authors with the permission of the American College of Surgeons—Commission on Cancer. Requests to access the datasets should be directed to https://www.facs.org/quality-programs/cancer/ncdb.

## Author Contributions

LH—investigation, methodology, writing—original draft, and writing—review and editing. AH—investigation, methodology, writing—original draft, and writing—review and editing. GC—conceptualization, formal analysis, investigation, methodology, writing—original draft, and writing—review and editing. AA—formal analysis, writing—original draft, and writing—review and editing. T-YT—writing—review and editing. LO—writing—review and editing. NP — formal analysis, methodology, and writing—review and editing. DB—writing—review and editing. JS—writing—review and editing. AM—writing—review and editing. ES—writing—review and editing. MH—writing—review and editing. JB-S—conceptualization, formal analysis, investigation, methodology, writing—original draft, and writing—review and editing. RL—conceptualization, formal analysis, funding acquisition, investigation, methodology, project administration, resources, supervision, writing—original draft, and writing—review and editing. All authors contributed to the article and approved the submitted version.

## Funding

This work was supported by University Hospitals Seidman Cancer Center and Case Comprehensive Cancer Center.

## Author Disclaimer

The published content and conclusions are the responsibility of the authors and does not represent the views of The American College of Surgeons and the American Cancer Society.

## Conflict of Interest

The authors declare that the research was conducted in the absence of any commercial or financial relationships that could be construed as a potential conflict of interest.

## Publisher’s Note

All claims expressed in this article are solely those of the authors and do not necessarily represent those of their affiliated organizations, or those of the publisher, the editors and the reviewers. Any product that may be evaluated in this article, or claim that may be made by its manufacturer, is not guaranteed or endorsed by the publisher.
